# Nestin Promotes Peritoneal Fibrosis by Protecting HIF1-α From Proteasomal Degradation

**DOI:** 10.3389/fphys.2020.517912

**Published:** 2020-12-16

**Authors:** Yangping Shentu, Huanchang Jiang, Xiaoyuan Liu, Hao Chen, Dicheng Yang, Jinqi Zhang, Chen Cheng, Yulin Zheng, Yang Zhang, Chaosheng Chen, Chenfei Zheng, Ying Zhou

**Affiliations:** ^1^Department of Pathology, The First Affiliated Hospital of Wenzhou Medical University, Wenzhou, China; ^2^Department of Internal Medicine, The First Affiliated Hospital of Wenzhou Medical University, Wenzhou, China; ^3^Guanghua School of Stomatology, Sun Yat-sen University, Guangzhou, China; ^4^Department of Nephrology, The First Affiliated Hospital of Wenzhou Medical University, Wenzhou, China

**Keywords:** peritoneal dialysis, peritoneal fibrosis, angiogenesis, HIF1-α pathway, nestin

## Abstract

**Background:**

Peritoneal dialysis (PD) is a treatment for end stage renal disease patients, but it can also cause peritoneal fibrosis. Nestin is known as a neural stem cell marker and it has many functions. The hypoxia induced factor (HIF) signaling pathway can be activated under hypoxia conditions, leading to the overexpression of some angiogenesis related genes. The aim of our study is to demonstrate Nestin’s role in the development of peritoneal fibrosis (PF), and to provide a new target (Nestin) to treat PF.

**Methods:**

PD mice models were constructed by an intraperitoneal administration of PDS at 10 ml/100g/d for 4 weeks. Nestin-positive cells were isolated from peritonea of Nestin-GFP mice by flow cytometry. The relationship of Nestin and HIF1-α-VEGFA pathway was detected by Nestin knockdown, Co-immunoprecipitation and immunofluorescence. Also, proteasomal activity was demonstrated by CHX and MG132 application, followed by Western blotting and Co-immunoprecipitation.

**Results:**

In our experiments, we found that Nestin expression resulted in PF. Also, HIF1-α/VEGFA pathway was activated in PF. Nestin knockdown reduced the level of HIF1-α. Nestin directly bound to HIF1-α and protected HIF1-α from proteasomal degradation. Overexpression of HIF1-α reverts the fibrosis levels in Nestin-knockdown cells. In brief, Nestin inhibited the degradation of HIF1-α by mitigating its ubiquitination level, leading to the activation of HIF1-α signaling pathway, and eventually promoted PF.

**Conclusion:**

We found a novel mechanism of PF that Nestin promotes by protecting HIF1-α from proteasomal degradation. Taken together, our key findings highlight a novel mechanism by which the silencing of Nestin hinders HIF1- α -induced PF.

## Introduction

Peritoneal dialysis (PD) is one of the most important renal replacement therapies for end-stage renal disease patients, which uses the peritoneal membrane as a dialysis membrane (Mehrotra et al., 2016). Peritoneal dialysis accounts for about 15% of end-stage renal disease patients (Li et al., 2017). During long term peritoneal dialysis, the peritoneal membrane is continually exposed to hyperosmotic, hyperglycemic, and acidic dialysis solutions, as well as mechanical stress (Bozkurt et al., 2008; Loureiro et al., 2013). One of the common and serious adverse effects of peritoneal dialysis is peritoneal fibrosis (PF). After a period stimulation, the peritoneal mesothelial cell will lose its normal structure, function and morphology (Yáñez-Mó et al., 2003), leading to peritoneal fibrosis. However, the mechanism of peritoneal fibrosis (PF) remains unclear.

As we know, HIF-1α is a key factor in the HIF signaling pathway (Semenza, 2012). Under normal oxygen conditions, HIF-1α is continually synthesized and degraded, which is triggered by the von Hippel-Lindau tumor-suppressor protein (VHL), the ligand-recognizing component of the E3 ubiquitin 2/elongin B & C/Rbx-1 (RING-box protein 1) complex, causing the ubiquitination and eventual degradation of HIF-1α. Under hypoxia conditions, HIF-1α degradation is inhibited, then the protein accumulates, dimerizes with HIF-1β (Liang et al., 2009) and the nucleus heterodimer activates its downstream pathways and eventually promotes the expression of angiogenesis related genes such as vascular endothelial growth factor (VEGF), erythropoietin (EPO), and platelet derived growth (PDGF) (Fábián et al., 2016). Moreover, plenty of research has shown that angiogenesis plays an essential part in organ fibrosis. For example, Iyer et al. (2015) found that inhibition of angiogenesis activity significantly mitigated bleomycin-induced pulmonary fibrosis. However, the relationship between HIF-1α and peritoneal fibrosis is still not demonstrated.

Nestin is a class VI intermediate filament protein, which was first used as a marker for stem cells in the developing central nervous system (Mignone et al., 2004). As we all know, the intermediate filament superfamily (IFs) is the most special in the cytoskeleton network system. In contrast with microfilament and microtubule, as the important housekeeping proteins, IFs are only expressed and regulated in a tissue, differentiation, and context-specific fashion, and are especially closely related with stem cells and cancers. In recent years, some reports showed that besides neural stem cells, other cells like mesenchymal like cells and even cancer cells also expressed Nestin (Day et al., 2007; Jiang et al., 2014). However, researchers recently proved that Nestin was not only a marker, but also had some other functions. For example, Yamagishi et al. (2019) found that Nestin was correlated with cancer cell metastasis; Reimer et al. (2009) showed that the expression of Nestin coincided with the accumulation of the glucocorticoid receptor. In this study, we aimed to find the role of Nestin in peritoneal fibrosis and the mechanism, thus providing a new target for the treatment of peritoneal fibrosis. We showed that Nestin expression had a close relationship with HIF-1α-VEGFA pathway activity. Our findings contribute to widening our understandings of how Nestin regulates peritoneal fibrosis in these proliferating cells.

## Materials and Methods

### Mouse Models

Animal protocols were approved by the Ethical Committee of Sun Yat- sen University. C57BL/6 mice (12 weeks old) were purchased from the Guangdong Medical Laboratory Animal Center (Guangzhou, China). Nestin-GFP mice were provided by Andy Peng Xiang. PD mice model was constructed by an intraperitoneal administration of PDS (4.25% DIANEAL; Deerfield, IL, Baxter, United States) at 10 ml/100g/d for 4 weeks. Control group was injected with PBS.

### Preparation of Cell Suspensions

Mice were killed by cervical dislocation. Peritoneal cells were obtained by injecting 5-10 ml RPMI 1640, supplemented with 10% FCS, glutamine, 2-ME, into the peritoneal cavity, gently agitating, and withdrawing medium aseptically with a Pasteur pipette. Cells were washed once with PBS. Samples that contained contaminating erythrocytes were discarded. Nestin-GFP cells from cell suspension were sorted by flow cytometry (BD Influx).

### Culture of Cells From Peritonea

Both Nestin-positive cells (Nes + cells) and Nestin-negative cells (Nes- cells) sorted from peritonea of Nestin-GFP mice were cultured. Transforming growth factor β1 (TGF-β1, MedChemExpress, NJ, United States) was used to stimulate the Nes + cells at 5 ng/mL for 48 h.

### Immunofluorescence

For immunofluorescent staining, cells attached to the glass slides or tissue sections harvested were fixed for 20 min with 4% paraformaldehyde (PFA), permeabilized in 0.1% Triton X-100 for 10 min, blocked for 30 min with PBS-3% BSA and finally incubated with proper primary and secondary antibodies in the dark. Nuclei visualization was subjected to DAPI staining for 3 min. Images were acquired under the fluorescence microscopy or using an LSM880 confocal microscope (Zeiss) and N-SIM super resolution microscopy (Nikon, Shinagawa, Japan) (Ma et al., 2019).

### Immunohistochemistry

Paraffin-embedded tissue sections (3 μm) were subjected to immunostaining using an UltraSensitiveTM SP (Mouse/Rabbit) IHC Kit (MXB). Each section was deparaffinized, treated with 3% H_2_O_2_ for 15 min, microwaved in 10 mM citric sodium (pH 6.0) for 15 min, incubated with the primary antibody overnight at 4°C, and then incubated with a secondary antibody for 30 min at 37°C. Signal amplification and detection was performed using the DAB system according to the manufacturer’s instructions (MXB) (Ma et al., 2019).

### Co-immunoprecipitation

For immunoprecipitation assays, cells were lysed using Pierce IP lysis buffer (Thermo Fisher Scientific, Waltham, MA, United States) supplemented with protease inhibitor cocktail (Roche). The cell lysates were centrifuged, and then immunoprecipitated overnight at 4°C using the indicated primary antibodies followed by incubation with Dynabeads Protein G (Life Technologies, Carlsbad, CA, United States) for 1 h. The immunocomplexes were washed twice with IP lysis buffer before being resolved by SDS-polyacrylamide gel electrophoresis (SDS-PAGE) and immunoblotted with indicated antibodies (Ma et al., 2019).

### Western Blotting

For Western blotting, cells were collected and lysed in 1 × RIPA buffer. After centrifugation at 15,000 × *g* for 5 min at 4°C, we collected the supernatant as the protein lysate. Protein samples were separated by 10% SDS-PAGE, transferred to a 0.45-μm pore-sized polyvinylidenedifluoride (PVDF) membrane (Millipore). The membranes were blocked with 5% BSA and then incubated with certain primary and secondary antibodies (Ma et al., 2019).

### Compounds

Translational inhibitor cycloheximide (CHX) and broad-spectrum proteasome inhibitor, MG132 were purchased from Sigma-Aldrich. Both compounds were dissolved in DMSO as 1000X stocks and were used at these final concentrations: CHX (50 μg/ml, 4 h); MG132 (20 μM, 6 h).

### RNA Isolation and Reverse Transcription PCR

Total RNA was extracted using a RNeasy mini kit (Qiagen, Hilden, Germany) according to the manufacturer’s protocol. Reverse transcription reactions were performed using murine leukemia virus reverse transcriptase and oligo-dT primers (Fermentas, Vilnius, Lithuania). Conventional PCR was performed using LC Taq (Fermentas, Vilnius, Lithuania) (Ma et al., 2019).

### Vectors and Reagents

For knockdown of Nestin expression, retrovirus vectors (pSM2) encoding shRNAs were purchased from Open Biosystems (Huntsville, AL, United States) (Yang et al., 2017). In addition, we used lentivirus provided by Andy Peng Xiang to upregulate Nestin. Myc-tagged Nestin was constructed using Invitrogen’s Gateway System. For overexpression of HIF1-α, plasmid was constructed using Invitrogen’s Gateway System. MG132 and CHX were purchased from Sigma. Non-target control (NTC) was used as the control group.

### Statistical Analysis

All data were presented as the mean ± S.E.M. from at least three independent experiments. One-way analysis of variance (ANOVA) was used to compare mean responses among the treatments. SPSS Version 14.0 (SPSS Inc., Chicago, United States) was used for all analyses. *P*-value < 0.05(^∗^) was considered significant (Huang et al., 2019).

## Results

### Nestin Expression Increases in PF

**FIGURE 1:**
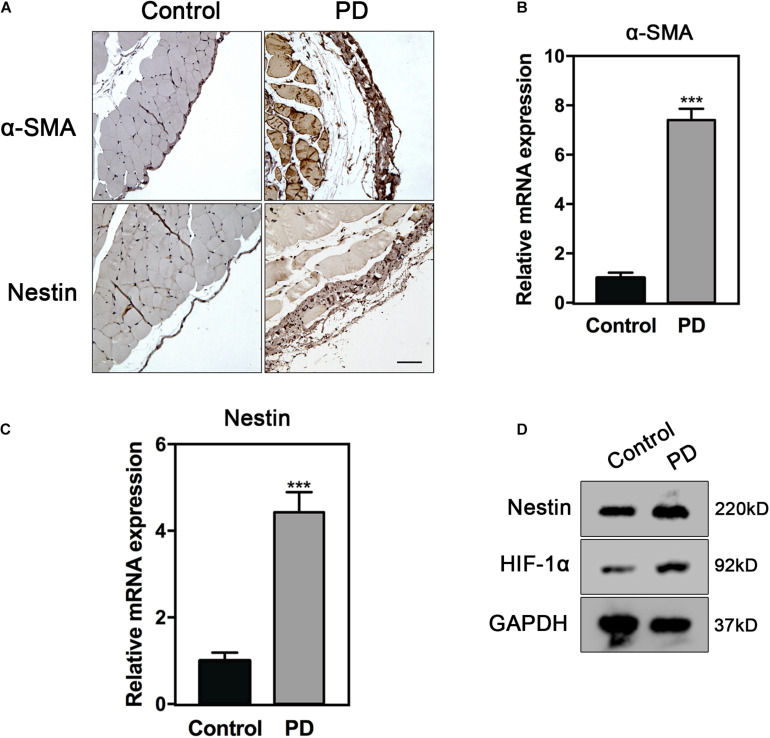
Nestin expression increases in PF. **(A)** IHC analysis of Nestin in control group or PD model group. Bar = 100 μm. Magnification, ×200. **(B)** qPCR analysis of α-SMA in control group or PD model group. **(C)** qPCR analysis of Nestin in control group or PD model group. **(D)** Immunoblotting analysis of HIF1-α and Nestin in control group or PD model group. ***p < 0.001.

To identify the mechanism of Nestin to PF, we constructed PD models to induce PF *in vivo*. α-smooth muscle actin (α-SMA), a common marker of myofibroblasts, was strongly expressed in the PD model group. The immunohistochemistry (IHC) staining results showed that the expression of α-SMA and Nestin increased in the PD model group (Figure 1A). In addition, the mRNA levels of α-SMA and Nestin also showed a significant increase in the PD model group (Figures 1B,C). Taken together, these data indicated that the increase of Nestin expression may be parallel to PF development.

### HIF1-α-VEGFA Pathway Is Up-Regulated in PF

**FIGURE 2:**
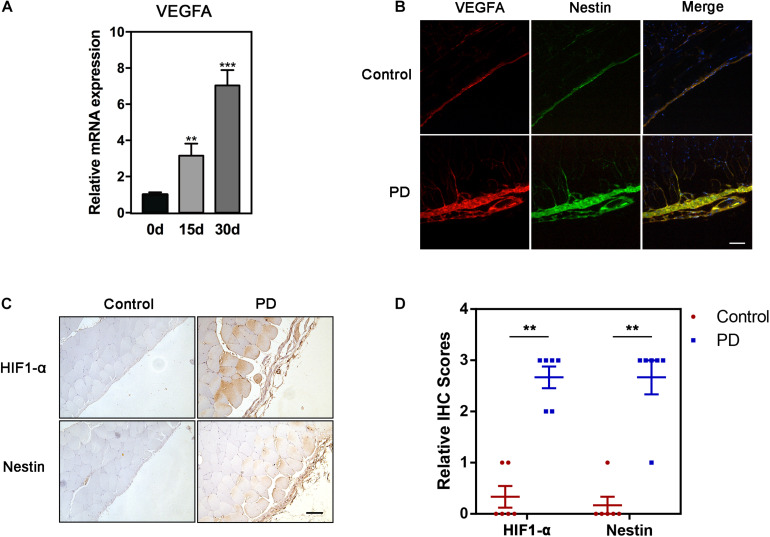
HIF1-α-VEGFA pathway is up-regulated in PF. **(A)** qPCR analysis of mRNA level of VEGFA in fibrotic peritonea. **(B)** If analysis of Nestin and VEGFA in control group or PD model group. Bar = 100 μm. Magnification, ×200. **(C)** IHC analysis of HIF1-α and Nestin in control group or PD model group. Bar = 100 μm. Magnification, ×200. **(D)** The quantification of HIF-1 and Nestin staining intensity. *p < 0.05, **p < 0.01.

The mRNA levels of VEGFA progressively increased in PD model on days 0, 15, and 30 (Figure 2A). In order to illustrate the correlation between Nestin and VEGFA during PF, we carried out the immunofluorescence (IF) experiment, showing increased expression of Nestin and VEGFA in the PD model group and co-localization of Nestin and VEGFA (Figure 2B). Since HIF1-α is commonly considered the upstream of VEGFA, we hypothesized that HIF1-α levels also increased in PF development. Furthermore, the IHC and immunoblotting experiment confirmed our hypothesis (Figures 2C,D). Together, these results indicated that Nestin-positive may participate in the development of PF.

### The Changes of Nestin Expression Regulate PF

**FIGURE 3:**
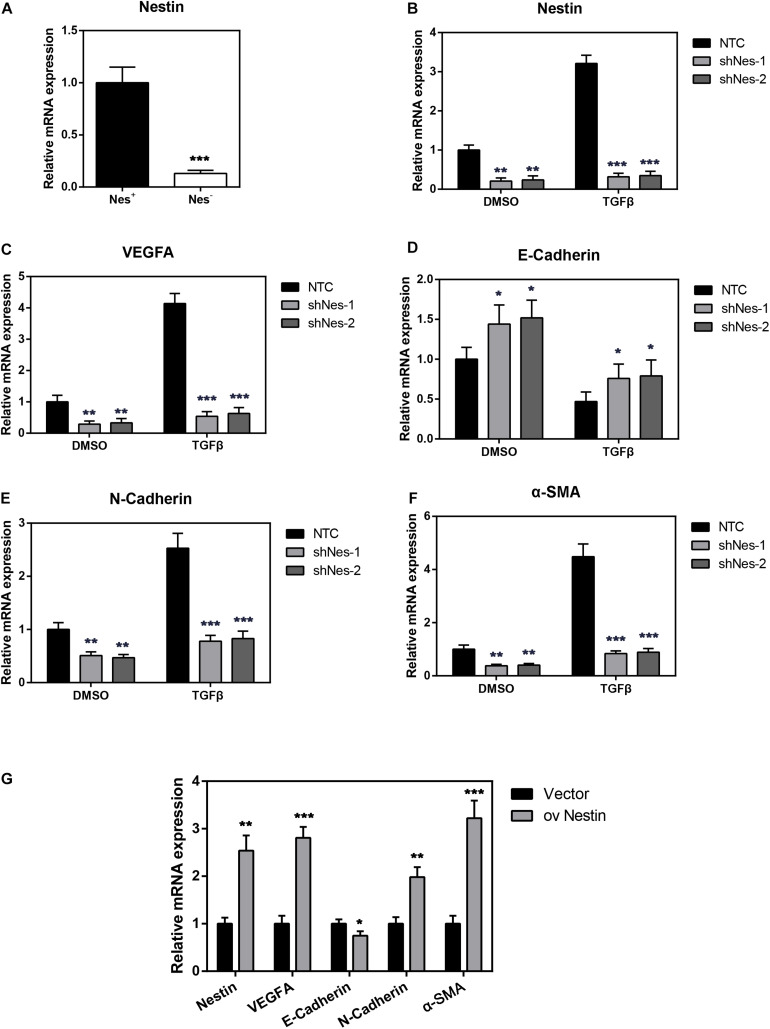
Nestin knockdown alleviates PF. **(A)** qPCR analysis of Nestin in both Nes + cells and Nes- cells. **(B)** qPCR analysis of the efficiency of Nestin knockdown in Nes + cells with TGF-β1 or not. **(C)** qPCR analysis of VEGFA following Nestin knockdown. **(D)** qPCR analysis of E-Cadherin following Nestin knockdown. **(E)** qPCR analysis of N-Cadherin following Nestin knockdown. **(F)** qPCR analysis of α-SMA following Nestin knockdown. **(G)** qPCR analysis of Nestin, VEGFA, E-Cadherin, N-Cadherin, and α-SMA following Nestin up-regulation. *P < 0.05, **P < 0.01, ***P < 0.001.

In order to investigate the relationship between Nestin and PF, we then both cultured Nes + cells and Nes- cells sorted from peritonea of Nestin-GFP mice. In order to show the up-regulation of Nestin in the Nes + cells, we performed q-PCR assay to compare the Nestin mRNA levels in Nes + cells and Nes- cells (negative control). The result showed that Nes + cells exhibited much higher expression of Nestin mRNA (Figure 3A). In addition, we both knock down and upregulate Nestin in Nes + cells. Meanwhile, we evaluated the expression of VEGFA by quantitative PCR (qPCR) in Nestin-knockdown cells, NTC, and Nestin-up-regulation cells, found that Nestin knockdown significantly decreased the mRNA levels of VEGFA (Figure 3C). As shown in Figures 3D–F, the mRNA levels of E-Cadherin increased and the mRNA levels of N-Cadherin decreased following Nestin knockdown, which indicated that Nestin knockdown reduced EMT change. Furthermore, we used lentivirus to upregulate Nestin, However, these changes could be reversed by Nestin up-regulation (Figure 3G).

We further detected the expression of Nestin, VEGFA, as well as fibrotic markers of Nes + cells in response to TGF-β1 stimulation. We found that TGF-β1 could remarkably enhance the expression of Nestin, VEGFA, α-SMA and EMT marker N-Cadherin, meanwhile inhibit the expression of E-Cadherin (Figures 3B–F). These expression patterns could be reversed by Nestin knockdown.

### Nestin Knockdown Decreases the Levels of HIF1-α

**FIGURE 4:**
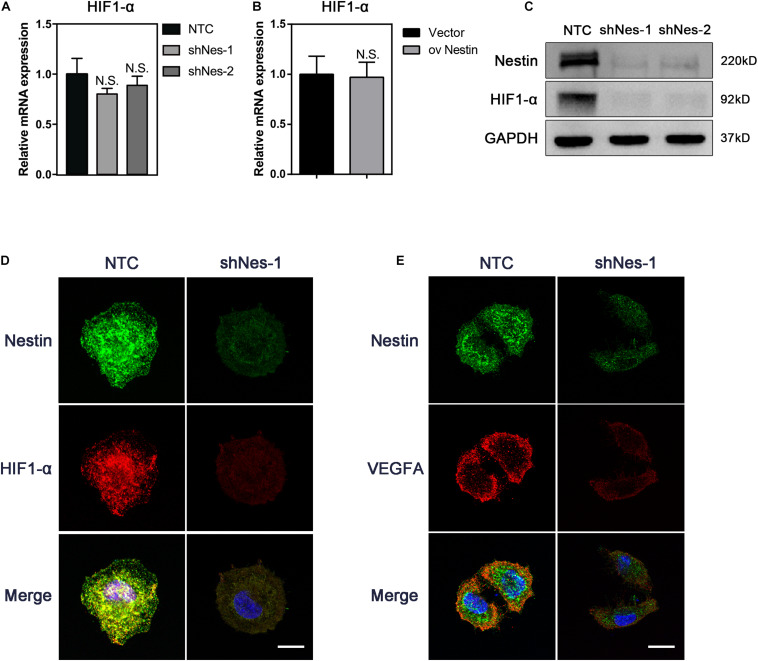
Nestin knockdown decreases the levels of HIF1-α. **(A)** qPCR analysis of HIF1-α mRNA levels following Nestin knockdown. **(B)** qPCR analysis of HIF1-α mRNA levels following Nestin up-regulation. **(C)** Immunoblotting analysis of HIF1-α protein levels following Nestin knockdown. **(D)** IF analysis of HIF1-α and Nestin following Nestin knockdown. Bar = 20 μm. Magnification, ×630. **(E)** IF analysis of VEGFA and Nestin following Nestin knockdown. Bar = 20 μm. Magnification, ×630.

To address this issue, we knocked down Nestin in the above-mentioned Nes + cells by shRNA. qPCR, immunoblotting, and IF were carried out to detect the expression of HIF1- α following Nestin knockdown. Compared with the control group, knockdown of Nestin did not affect the mRNA levels of HIF1-α but significantly decreased the protein levels of HIF1-α (Figures 4A–C). IF suggested a decreased expression of VEGFA, the downstream of HIF1-α (Figures 4D,E). Together, these data demonstrated that Nestin deficiency could down-regulate HIF1-α-VEGFA pathway.

### Nestin Interacts With HIF1-α

**FIGURE 5:**
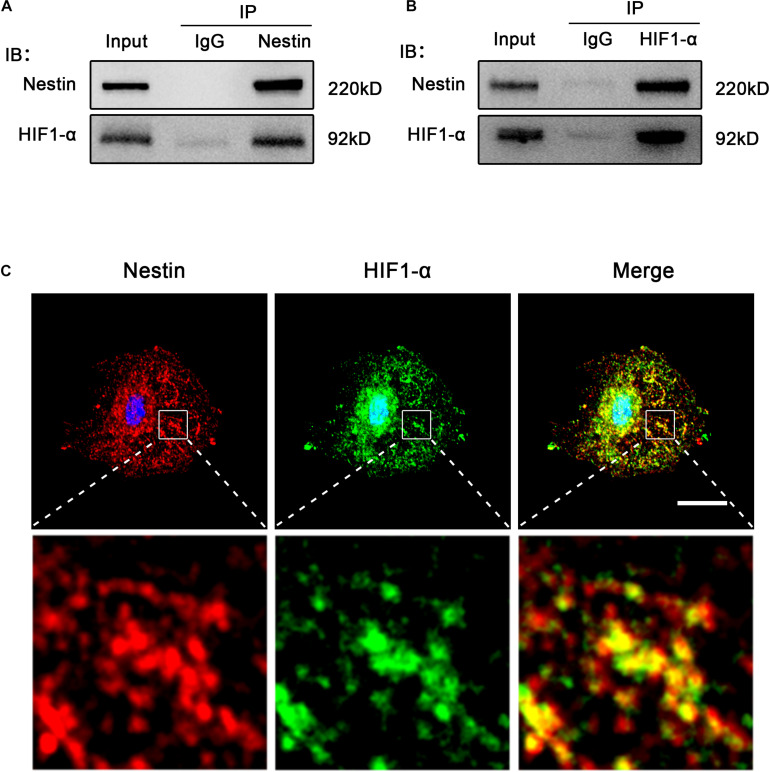
Nestin interacts with HIF1-α. **(A)** Co-immunoprecipitation of Myc-Nestin and HIF1- α from Nes + cell. IgG was used as a negative control for the immunoprecipitation. **(B)** Co-immunoprecipitation of endogenous HIF1- α and Nestin from Nes + cell lysates with anti- HIF1-α antibody. **(C)** IF staining of Nestin and HIF1-α in Nes + cells. Bar = 20 μm. Magnification, ×630.

To test this possibility, we detected the relationship between Nestin and HIF1-α. As shown in Figure 5A, HIF1 co-precipitated with Nestin. We observed a direct interaction between HIF1- α and Nestin by reciprocal immunoprecipitation with HIF1- α antibody (Figure 5B). Furthermore, super resolution microscope showed that Nestin and HIF1-α were co-localized in Nes + cells (Figure 5C). Together, these data demonstrated that there is a direct interaction between HIF1-α and Nestin.

### Nestin Protects HIF1-α From Proteasomal Degradation

**FIGURE 6:**
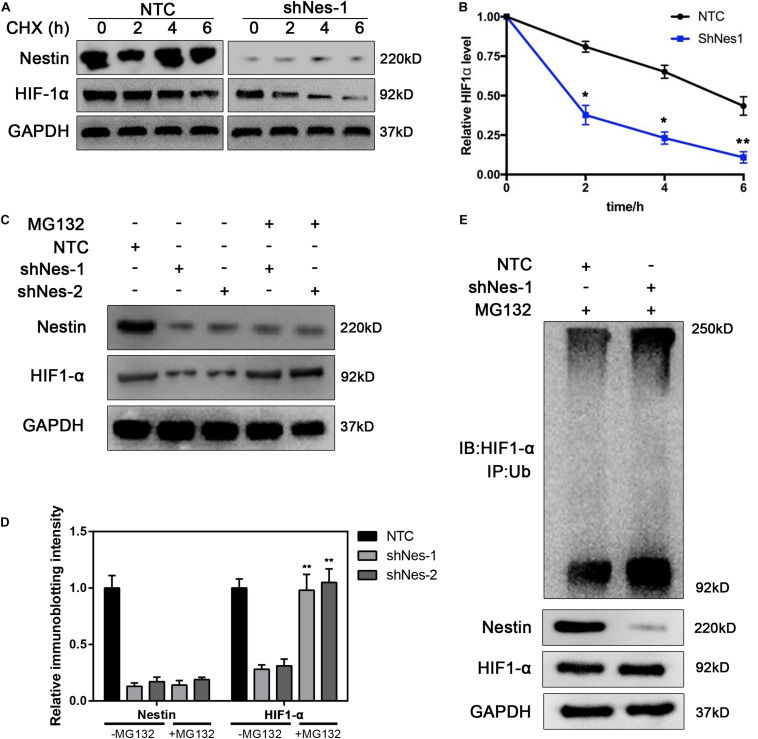
Nestin protects HIF1-α from proteasomal degradation. **(A,B)** Half-life analysis of HIF1-α in control and Nestin-knockdown cells. Both groups were treated with cycloheximide (50 μg/ml), harvested at the indicated times, and subjected to immunoblotting **(A)**. Quantification of HIF1-α levels relative to GAPDH is shown **(B)**. **(C)** Identification of the pathway responsible for HIF1-α degradation upon Nestin knockdown. Control and Nestin-knockdown cells were treated with MG132 (20 μM, 6 h), and then proteins were extracted and subjected to immunoblotting. **(D)** The quantification of HIF-1 and Nestin immunoblotting intensity. **(E)** The effects of Nestin knockdown on ubiquitination of HIF1-α were analyzed by *in vivo* ubiquitination assays. HIF1-α was immunoprecipitated with anti-HIF1-α antibody and immunoblotted with anti-Ub antibody. The quantified results were presented as mean ± SEM of three independent experiments, as assessed using two-way ANOVA test. **P < 0.01.

To further investigate the relationship between Nestin and HIF1-α, the expression of HIF1-α was detected in NTC and Nestin-knockdown cells treated with the translational inhibitor cycloheximide (CHX). Compared to controls, HIF1-α was more highly degraded in Nestin-knockdown cells by immunoblotting (Figure 6A), and HIF1-α had a shorter half-life in Nestin-knockdown cells (Figure 6B). These results demonstrated that Nestin affected HIF1-α stability *per se*. To further elucidate the mechanism of Nestin-knockdown-induced degradation of HIF1-α, pretreatment with the broad-spectrum proteasome inhibitor, MG132, yielded an almost complete recovery of HIF1-α in Nestin-knockdown cells (Figures 6C,D). This indicated that Nestin deficiency increased HIF1-α degradation through a proteasomal pathway. To further confirm this, we examined the levels of HIF1- α ubiquitination in Nestin-knockdown and control cells (Figure 6E). We found that Nestin knockdown increased the ubiquitination of HIF1-α. Thus, Nestin regulated the stability of HIF1-α by protecting it from proteasomal degradation.

### Overexpression of HIF1-α Reverts the Fibrosis Levels in Nestin-Knockdown Cells

**FIGURE 7:**
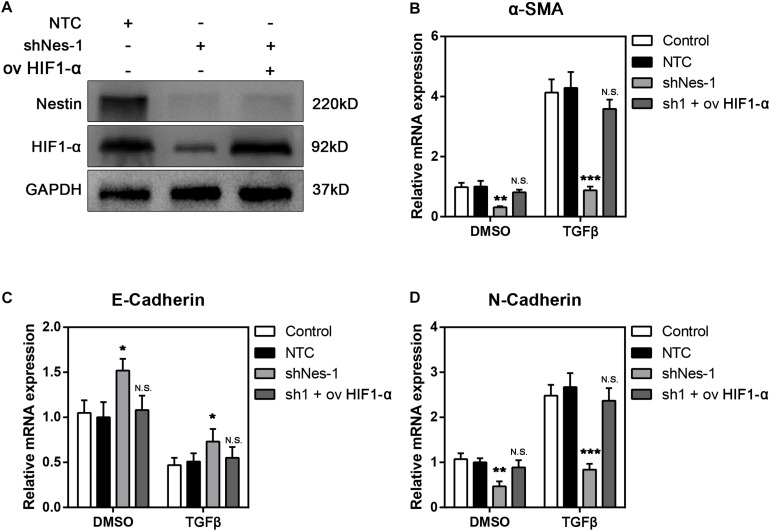
Overexpression of HIF1-α reverts the fibrosis levels in Nestin-knockdown cells with TGF-β1 or not. **(A)** Immunoblotting analysis of the efficiency of HIF1-α overexpression following Nestin knockdown. **(B)** qPCR analysis of α-SMA following HIF1-α rescue. **(C)** qPCR analysis of E-Cadherin following HIF1-α rescue. **(D)** qPCR analysis of N-Cadherin following HIF1-α rescue.

We next investigated the possibility of targeting HIF1-α to alleviate fibrosis. We overexpressed HIF1-α in Nestin- knockdown cells. The immunoblotting experiment proved the efficiency of HIF1-α overexpression (Figure 7A). The qPCR experiment showed that overexpression of HIF1-α canceled the alleviating of fibrosis following Nestin-knockdown (Figure 7B). Furthermore, we also found that the mRNA levels of E-Cadherin decreased, and the mRNA levels of N-Cadherin increased in the overexpressed HIF1-α group (Figures 7C,D). Together, these results suggested that Nestin knockdown alleviates fibrosis by decreasing the protein levels of HIF1-α.

## Discussion

PF is a common pathological change during long-term PD patients, which also weakens the therapeutic effect of PD (Grassmann et al., 2005). However, the mechanism of PF remains unclear. In our study, we demonstrate the positive correlation between Nestin and PF. With further study, we show that Nestin can mitigate the ubiquitination and degradation of HIF1-α and activate the HIF signaling pathway, eventually promoting the expression of VEGFA. In a word, we find the relationship between Nestin-HIF1-α-VEGFA pathway in PF.

Nestin is a type VI intermediate filament protein, which is widely used as a marker for neural stem cells and progenitor cells in the central nervous system. Nestin participates in the assembly of other intermediate filaments and plays a vital role in remodeling of the cell together with other structural proteins. In recent years, plenty of studies have projected that Nestin is essential in regulating signal pathways. For example, Zhao et al. showed that Nestin can positively regulate the Wnt/β-catenin pathway (Zhao et al., 2014); Li et al. found that Nestin can activate the Hedgehog pathway (Li et al., 2016). Meanwhile, Xiang et al. found that during the embryonic development of mice, the ability of angiogenesis was greatly reduced in Nestin-knockout group (Wang et al., 2018). Also, Nestin expression is upregulated in numerous organ fibrosis (Soler-Torronteras et al., 2014; Cao-Sy et al., 2019). In this study, we demonstrate that Nestin can protect the degradation of HIF1-α by directly binding to it.

The HIF1-α signaling pathway contains two key factors, HIF-α (HIF-1α, HIF-2α, HIF-3α) and HIF-1β. The oxygen level will change the amount of HIF-1α but the protein level of HIF-1β is not influenced. Under normal oxygen states, HIF-1α is hydroxylated by the prolyl hydroxylase domain (PHD) family (Kaelin, 2005), providing a binding site for the von Hippel-Lindau (VHL) protein, which is a component of a ubiquitin ligase complex. As a result, HIF-1α is polyubiquitinated and leaded to proteasomal degradation. Under a hypoxia state, HIF-1α accumulates, dimerizes with a HIF-1β, translocates to the nucleus, and transcriptionally activates its downstream genes (Kaelin and Ratcliffe, 2008). Previous studies have shown that HIF1-α-VEGFA pathway is involved in various organ fibrosis (Barratt et al., 2017; Elzamly et al., 2017; Li et al., 2019). In this study, we found that Nestin can interact with HIF-1α and increase its protein level. With further study, we showed that Nestin can alleviate the ubiquitination level and prevent HIF-1α degradation but does not affect the synthesis of HIF-1α.

The downstream of HIF1-α signaling pathway contains a lot of genes, including genes involved in anaerobic metabolism (Melstrom et al., 2011), DNA damage responses (Sendoel et al., 2010) and so on. Besides, several recent studies also show that EMT is closely related to HIF1-α signaling pathway (Yang et al., 2015). During the EMT process, the epithelial cell markers decrease, the mesenchymal cell markers increase and cell to cell adhesion is lost. Additionally, EMT is regulated by several transcription factors like Snail, Twist, Slug (Song et al., 2017). Research has shown that HIF1-α can induce EMT by regulating Twist. HIF signaling pathway contributes to angiogenesis as well, which activates the expression of angiogenesis related genes like VEGFA and EPO (Fábián et al., 2016). In our experiment, the overexpression of Nestin activates HIF1-α signaling pathway and promotes the EMT process and angiogenesis, which eventually leads to PF.

PF is caused by long-term PD. The stimulation and damage of the procedure leads to peritoneal pathological changes. It is widely accepted that when the peritoneum is undergoing the fibrosis process, the peritoneal mesothelial cell layer will go through the epithelial to mesenchymal transformation (EMT) (Selgas et al., 2006). During the EMT process, cells go through the loss of epithelial cell markers like E-cadherin, cytokeratin and gain mesenchymal cell markers like N-cadherin and α-SMA. The adhesion between cells is also lost (De Craene and Berx, 2013). Besides the phenotype changes, cells undergoing EMT also upregulate some key transcription factors like Snail (Cano et al., 2000) and Twist (Yang et al., 2004). With these cellular changes, the peritoneal epithelial cells transform into myofibroblast cells and promote PF. In our experiment, the knockdown of Nestin reduced the mRNA levels of EMT related genes, suggesting that Nestin could regulate the EMT process. On the other hand, the downstream of HIF1-α signaling pathway also contains lots of genes, including genes involved in anaerobic metabolism (Melstrom et al., 2011), DNA damage responses (Sendoel et al., 2010) and so on. Importantly, several recent studies also show that EMT is closely related to HIF1-α signaling pathway (Yang et al., 2015). Research has shown that HIF1-α can induce EMT by regulating Twist, and HIF signaling pathway contributes to angiogenesis as well, which activates the expression of angiogenesis related genes like VEGFA and EPO (Fábián et al., 2016). In our experiment, After Nestin knockdown, the HIF1-α signaling pathway became inactivated, which eventually leads to the inhibition of PF.

## Conclusion

In conclusion, we showed that Nestin expression is related to HIF1-α/VEGFA pathway activity in PF. Nestin protected the proteasomal degradation of HIF1-α, resulting in the activation of HIF1-α-VEGFA signaling pathway, and eventually promoted PF. Our results shed light on a novel mechanism of Nestin in HIF1- α -induced PF.

## Data Availability Statement

The datasets generated for this study are available on request to the corresponding author.

## Ethics Statement

The animal study was reviewed and approved by the Ethical Committee of Sun Yat-sen University.

## Author Contributions

YS, HJ, and JZ performed the experiments and analyzed the data. YS, DY, YiZ, and CZ participated in manuscript preparation. YS, HJ, JZ, YuZ, and YiZ detection of immunofluorescence, immunohistochemistry, western blot, and qPCR. JZ and YS detection of co-immunoprecipitation. CC, CZ, and YaZ analyzed and interpreted the data. YS, CZ, and YiZ participated in the experimental design and coordination and wrote the manuscript. CZ, CC, and YiZ influenced the project, supervised the experiments, and analyzed data. All authors contributed to the article and approved the submitted version.

## Conflict of Interest

The authors declare that the research was conducted in the absence of any commercial or financial relationships that could be construed as a potential conflict of interest.
